# Teacher–child relationships and preschool peer acceptance: a two wave longitudinal study of theory informed indirect pathways through emotion understanding and social initiative

**DOI:** 10.3389/fpsyg.2026.1809721

**Published:** 2026-10-07

**Authors:** Yuebo Li, Haowen Dang, Yong Yang, Jianye Li

**Affiliations:** 1 Faculty of Education, Taiyuan Normal University, Jinzhong, China; 2 Faculty of Education, University Putra Malaysia, Serdang, Selangor, Malaysia; 3 Department of Physical Education, Shanxi Medical University, Jinzhong, China

**Keywords:** emotion understanding, peer acceptance, social initiative, teacher–child relationships, theory-informed indirect pathway, two-wave longitudinal design

## Abstract

**Introduction:**

Preschool peer acceptance is a pivotal marker of social-emotional well-being and future adaptation. Although teacher–child relationship quality is a known correlate of children’s peer status, the mechanisms linking this vertical relationship to horizontal peer relations remain insufficiently understood.

**Methods:**

This two-wave longitudinal study examined whether emotion understanding and social initiative were associated with the prospective link between teacher–child relationship quality and later peer acceptance. Data were collected from 319 Chinese preschool children across a 12-month interval. Teachers reported teacher–child closeness, conflict, and children’s social initiative; children completed an emotion understanding task; and peer acceptance was assessed using peer nominations. Structural equation models controlled for baseline levels of emotion understanding, social initiative, peer acceptance, and relevant covariates.

**Results:**

Results showed that T1 teacher closeness positively predicted, whereas teacher conflict negatively predicted, T2 peer acceptance. Emotion understanding and social initiative each served as significant indirect pathways linking teacher–child relationship quality with later peer acceptance. In addition, a theory-informed serial indirect pathway from T1 teacher–child relationship quality to T2 peer acceptance via T2 emotion understanding and T2 social initiative was statistically significant.

**Discussion:**

However, because the study was observational and because the mediators and outcome were all assessed at T2, the findings should be interpreted as prospective associations consistent with the proposed theoretical model rather than as evidence of causal or strictly temporal mechanisms. These findings suggest that supportive teacher–child relationships may be linked to peer acceptance through children’s socioemotional understanding and proactive peer engagement.

## 1 Introduction

The preschool years constitute a formative period for the development of social-emotional competence (Pahl and Barrett, 2007; Handayani and Kaffa, 2025). During this period, children gradually move from primarily vertical, attachment-based relationships with caregivers toward increasingly salient horizontal relationships with peers (Jia et al., 2025). Peer acceptance - the extent to which a child is liked, preferred, and sought out by classmates - is an important marker of this transition. It has been linked to concurrent and later developmental outcomes, including academic engagement, self-esteem, prosocial behavior, and psychological wellbeing, whereas peer rejection has been associated with loneliness, anxiety, and school disengagement (Ladd and Oden, 1979; La Greca, 1981). Recent work continues to emphasize the importance of early social-emotional development for school readiness and adjustment (Handayani and Kaffa, 2025). Nevertheless, the field still requires more precise accounts of the developmental processes through which classroom relationships are associated with peer acceptance.

The preschool classroom provides a proximal social context in which teacher-child and peer relationships are intertwined. Within this setting, young children interact daily with teachers and classmates, and these interactions provide repeated opportunities to learn, practice, and revise social behaviors (Booren et al., 2012). Prior research has shown that positive teacher-child relationships are associated with more favorable peer relations (Wang et al., 2016; Kiuru et al., 2015). More recent literature has also encouraged a broader view of preschool social development, suggesting that social functioning is connected not only with relational experiences but also with attention, learning-related engagement, and classroom participation (Commodari et al., 2026). These findings are useful for the present study because they indicate that links between teacher-child relationships and peer acceptance should be understood within a broader developmental system rather than as a single isolated pathway.

Attachment-oriented perspectives provide one theoretical basis for understanding these associations. The teacher-child relationship has been described as a classroom-based secure base that may support early school adaptation and social exploration (Verschueren and Koomen, 2012; Verschueren, 2015). Although teacher-child relationships are distinct from parent-child attachment relationships, warm, sensitive, and low-conflict interactions may provide children with a sense of emotional security in the classroom (Kennedy and Kennedy, 2004; Bergin and Bergin, 2009). Such security may reduce vigilance and anxiety, allowing children to engage more confidently with the surrounding social environment, including peers (Davies and Martin, 2013). Over time, children may internalize relational experiences and develop working models of the self and others (Bretherton et al., 1990). By contrast, relationships characterized by negativity, dependency, or frequent conflict may be associated with perceptions of threat, defensive responses, and difficulties in social exploration (Mikulincer et al., 2009). Consistent with this view, close teacher-child relationships have been linked to prosocial behavior, cooperation, and peer acceptance, whereas conflictual relationships have been linked to aggression, withdrawal, and peer rejection (Troop-Gordon and Kopp, 2011; Runions and Shaw, 2013).

Despite this evidence, existing studies often document associations without sufficiently specifying the child-level competencies through which teacher-child relationships are linked to peer outcomes. One candidate process is emotion understanding (EU). EU refers to children’s ability to recognize, label, and reason about the causes, consequences, and mentalistic antecedents of emotions in themselves and others (Brown and Dunn, 1996; Sprung et al., 2015). Social information-processing models suggest that successful peer interaction depends partly on the accurate encoding and interpretation of social cues, many of which are emotional in nature (Dodge, 2014; Lemerise and Arsenio, 2000). Teachers may function as emotion socializers through emotion talk, responses to children’s emotional displays, and everyday guidance in emotionally meaningful situations. Recent research has continued to highlight the importance of classroom experiences for children’s cognitive and emotional development (Cankaya et al., 2023; Kaizar and Alordiah, 2023). However, this literature also suggests that emotion understanding should not be treated as an isolated skill. It is likely embedded in broader attentional, language, and interactional contexts that shape how children perceive and respond to social information.

Longitudinal evidence indicates that positive teacher-child interaction quality is associated with later gains in emotion understanding, and children with stronger emotion understanding are often better liked by peers (Hu et al., 2018). Thus, the pathway linking teacher-child relationship quality, emotion understanding, and peer acceptance is theoretically plausible. At the same time, recent developmental work cautions against a narrow interpretation of this pathway. Commodari et al. (2026) found that preschool children’s social functioning profiles were closely associated with visual focused attention, visual-spatial focused attention, short-term attentional span, and learning-related outcomes. Their findings suggest that children’s social functioning reflects the interplay of relational, attentional, and learning-related competencies. This evidence is important for the present study because emotion understanding may depend not only on emotional knowledge but also on the child’s ability to attend to relevant cues, sustain engagement, and participate in structured classroom activities.

A second candidate process is social initiative (SI). In the present study, SI refers to the child’s tendency to proactively and positively initiate interaction with peers, such as approaching peers, inviting them to play, starting conversations, and entering group activities appropriately (Strain and Odom, 1986; Huber et al., 2019). The distinction between social competence and social performance is central here. Possessing socio-cognitive skills such as emotion understanding does not necessarily mean that children use those skills effectively in real peer contexts (Jurevièienë et al., 2012). Children who can read emotional and social cues may be better positioned to judge when and how to approach peers, but successful peer engagement also requires behavioral enactment. Prior work suggests that proactive peer engagement is associated with social opportunity and acceptance (Lam, 2012). Longitudinal peer influence research also indicates that peer processes unfold through complex and reciprocal patterns rather than simple unidirectional effects (Giletta et al., 2021). Therefore, SI may help clarify how socio-cognitive understanding is translated into observable peer interaction, while still being only one component of a broader social-developmental system.

Integrating these perspectives, the present study examined a theory-informed indirect pathway in which T1 teacher-child relationship quality was associated with T2 peer acceptance through T2 emotion understanding and T2 social initiative. This model was intended to capture a relational-cognitive-behavioral pattern of association: teacher-child relationship quality provides the classroom relational context; emotion understanding reflects a socio-cognitive capacity for interpreting emotional cues; and social initiative reflects the behavioral tendency to engage peers proactively. However, this pathway should not be interpreted as a definitive developmental sequence. Emotion understanding, social initiative, and peer acceptance were assessed at the same follow-up wave; therefore, the ordering among these T2 variables is theoretically specified rather than empirically demonstrated. In addition, recent evidence on attention and learning-related competencies indicates that the proposed mediators may be intertwined with broader developmental capacities that were not fully measured in the present study.

The present study addresses several gaps in the literature. First, it examines teacher-child relationship quality and peer acceptance in a two-wave longitudinal framework rather than relying solely on cross-sectional associations. Second, it considers both emotion understanding and social initiative, thereby linking socio-cognitive interpretation with behavioral engagement. Third, it includes baseline controls for the mediator and outcome variables, allowing the analyses to focus on prospective associations over a 12-month period. Nevertheless, the model remains observational and should be interpreted cautiously. It can identify predictive and indirect associations, but it cannot establish causality or fully capture reciprocal developmental processes.

Accordingly, the present study aimed to investigate the longitudinal interplay among teacher-child relationship quality, emotion understanding, social initiative, and peer acceptance in preschool children over 12 months. Based on the integrated theoretical framework, we proposed a hierarchy of hypotheses. First, we expected significant positive intercorrelations among teacher-child closeness, emotion understanding, social initiative, and peer acceptance, and negative correlations involving teacher-child conflict, both concurrently and across time (Hypothesis 0). Second, we hypothesized that T1 teacher-child closeness would positively predict, whereas T1 teacher-child conflict would negatively predict, residualized change in T2 peer acceptance after controlling for T1 peer acceptance and covariates (Hypothesis 1). Third, we expected T2 emotion understanding and T2 social initiative to independently mediate the association between T1 teacher-child relationship quality and T2 peer acceptance, controlling for baseline levels of the respective mediator and outcome (Hypothesis 2). Finally, we tested a theory-informed serial indirect pathway in which T1 teacher-child relationship quality was associated with T2 peer acceptance through T2 emotion understanding and T2 social initiative (Hypothesis 3). Because emotion understanding, social initiative, and peer acceptance were assessed at the same follow-up wave, this final hypothesis concerns a theoretically specified ordering rather than an empirically demonstrated temporal sequence among the T2 variables.

By testing this model, the study seeks to advance a more precise understanding of how teacher-child relationship quality is prospectively associated with preschool peer acceptance. The revised model emphasizes relational security, emotion understanding, and social initiative while also recognizing that these processes are embedded in broader attentional, learning-related, and classroom contexts. In this way, the study offers a theoretically grounded but appropriately cautious account of early peer adjustment and identifies potential targets for future multi-wave and intervention research.

## 2 Materials and methods

### 2.1 Participants

A total of 350 children (mean age at Time 1 = 4.52 years, SD = 0.71; 48% girls) from 12 public preschool classrooms in Taiyuan, China were initially recruited. Two-wave longitudinal data were collected with a 12-month interval. At Time 2 (T2), 319 children (mean age = 5.49 years, SD = 0.69; 47% girls) remained, resulting in a retention rate of 91.4%. Attrition analyses revealed no significant differences between children who remained and those who dropped out on any T1 study variables (*p*s > 0.10). An a priori power analysis was conducted using G*Power 3.1 to justify the required sample size for the primary predictive paths. Because G*Power does not directly estimate power for the full structural equation model with indirect pathways, we used the most parameterized regression equation in the structural model as an approximate basis for sample size planning. The analysis was conducted using the *F*-test family, linear multiple regression: fixed model, *R*^2^ deviation from zero. We assumed a small-to-medium effect size of Cohen’s *f*^2^ = 0.06, an alpha level of 0.05, desired statistical power of 0.80. The analysis indicated that a minimum sample size of approximately 300 participants was required. The final analytic sample of 319 children exceeded this requirement, yielding an estimated achieved power of approximately 0.83. Primary caregivers provided written informed consent, and the study protocol was approved by the Institutional Review Board of Shanxi medical university.

### 2.2 Procedure

The study employed a two-wave longitudinal design. At Time 1 (T1), during the fall semester, the following measures were administered in sequence: (a) Head teachers completed the Teacher-Child Relationship Scale and the Social Initiative Questionnaire for each participating child in their class. (b) Trained research assistants individually administered the Test of Emotion Comprehension to each child in a quiet room at the preschool. (c) Peer nominations were conducted individually and privately using a photo-roster method. (d) Parents completed a brief demographic questionnaire.

Approximately 12 months later, at Time 2 (T2), the same measures were readministered in an identical sequence, with the exception of the Teacher-Child Relationship Scale (which was only administered at T1 as the predictor variable). All research assistants underwent standardized training to ensure consistency in test administration and behavioral observation protocols.

Teacher–child relationship quality was assessed only at T1 because the present study was designed to examine whether the initial relational context in the preschool classroom was prospectively associated with later socio-emotional functioning and peer acceptance. Thus, T1 teacher–child closeness and conflict were treated as baseline relational predictors rather than as repeatedly measured developmental outcomes. Nevertheless, this design choice limited our ability to examine changes in teacher–child relationship quality or reciprocal associations between teacher–child relationships and children’s later peer adjustment.

### 2.3 Instruments

#### 2.3.1 Teacher-child relationship quality at T1

The Student-Teacher Relationship Scale (STRS) (Pianta, 2001) Short Form was used to assess teachers’ perceptions of their relationship with each child. We used the two core subscales: Closeness (11 items, e.g., “I share an affectionate, warm relationship with this child”) and Conflict (12 items, e.g., “This child and I always seem to be struggling with each other”). Teachers rated each item on a 5-point Likert scale (1 = Definitely does not apply, 5 = Definitely applies). Subscale scores were calculated as the mean of relevant items, with higher scores indicating greater closeness or conflict. In this sample, Cronbach’s α was .89 for Closeness and .91 for Conflict.

#### 2.3.2 Emotion understanding (mediator 1, T1 and T2)

Children’s emotion understanding was assessed using an adapted version of the Test of Emotion Comprehension (Pons and Harris, 2019), a widely used and validated individual task for preschoolers. The assessment comprises nine components tapping different aspects of EU (e.g., recognition, external cause, desire-based emotion). For this study, we selected four age-appropriate components (Recognition, External Cause, Desire, and Belief) comprising 12 items. For each item, the child was presented with a short vignette (supported by pictures) and asked to point to the correct facial expression among four options (happy, sad, angry, afraid). Example: “This girl just found her favorite toy under the bed. How does she feel?” (Point to the correct face). One point was awarded for each correct answer, yielding a total score ranging from 0 to 12. Parallel versions with different story content were used at T1 and T2 to minimize practice effects. Internal consistency (KR-20) was 0.78 at T1 and 0.81 at T2.

#### 2.3.3 Social initiative (mediator 2, T1 and T2)

Social initiative was assessed using a teacher-report scale developed for the present study to capture preschool children’s proactive and positive initiation of peer interaction. In this study, social initiative was conceptualized as a specific behavioral component of social functioning rather than as a global measure of social competence. Whereas broader social competence includes multiple domains such as cooperation, compliance, emotion regulation, prosocial behavior, conflict resolution, and peer status, social initiative refers specifically to children’s tendency to voluntarily approach peers, invite peers to participate in play, initiate conversations, appropriately enter group activities, and maintain positive engagement during peer interaction.

The initial item pool was generated by reviewing theoretically relevant dimensions of peer play, peer-entry behavior, and social approach, with reference to the Penn Interactive Peer Play Scale and the Child Social Preference Scale. Items were selected or adapted only when they directly reflected proactive peer engagement rather than broader aspects of social competence. An initial pool of 10 items was reviewed by three experts in early childhood education and developmental psychology. The experts evaluated each item for conceptual relevance, clarity, developmental appropriateness, and cultural suitability for Chinese preschool classrooms. Based on their feedback, redundant or overly broad items were removed, and the wording of several items was simplified. The final scale included six items.

Teachers rated each item on a 5-point scale ranging from 1 = never to 5 = always. Example items included: “This child often invites other children to play,” “This child starts conversations with peers during free play,” and “This child actively joins group play in an appropriate way.” Higher mean scores indicated greater social initiative.

In the present sample, the scale demonstrated excellent internal consistency, with Cronbach’s α = 0.86 at T1 and .88 at T2. Confirmatory factor analyses supported the proposed one-factor structure at both waves. The one-factor model showed excellent fit at T1, χ^2^(9) = 8.06, χ^2^/df = 0.90, CFI = 1.000, TLI = 1.001, RMSEA = 0.000, SRMR = 0.003, and at T2, χ^2^(9) = 10.90, χ^2^/df = 1.21, CFI = 0.999, TLI = 0.999, RMSEA = 0.027, SRMR = 0.005. Standardized factor loadings ranged from 0.745 to 0.859 at T1 and from 0.736 to 0.833 at T2. Composite reliability was 0.984 at T1 and 0.979 at T2, and average variance extracted was 0.910 at T1 and 0.886 at T2. These findings provided preliminary evidence for the internal consistency, structural validity, and convergent validity of the Social Initiative scale in the present sample.

#### 2.3.4 Peer acceptance (outcome, T1 and T2)

Peer acceptance was assessed using a standard peer nomination procedure (Cillessen and Marks, 2017). The procedure was conducted separately within each classroom because peer nominations are inherently classroom-referenced. Children were presented with a photo roster of all participating classmates in their classroom and were interviewed individually and privately by trained research assistants in a quiet area of the preschool. Before the nomination task, the researcher ensured that the child could recognize classmates from the photo roster. Children were not allowed to nominate themselves.

For each classroom, children were asked two questions: “Who do you like to play with the most?” and “Who do you like to play with the least?” Children were allowed to nominate an unlimited number of classmates for each question. The use of unlimited nominations was intended to reduce restriction in children’s responses and to provide a more complete representation of peer preference within each classroom.

The final analytic sample included children from 12 classrooms. Classroom size in the analytic sample ranged from 26 to 27 participating children per classroom, with an average classroom size of 26.58 children. Among children retained in the final longitudinal sample, usable peer nomination data were available at both waves. The overall longitudinal retention rate from the initially recruited sample was 91.4% because 319 of the 350 initially recruited children remained at T2. Thus, the peer nomination scores used in the main analyses were based on children with valid classroom-based nomination data across the two assessment waves.

For each child, the number of positive nominations received and the number of negative nominations received were counted within the classroom. To account for differences in classroom size and classroom-level nomination patterns, positive and negative nomination counts were standardized within each classroom. A social preference score was then calculated by subtracting the standardized negative nomination score from the standardized positive nomination score. Higher scores indicated greater peer acceptance. This classroom-standardized peer nomination method is widely used for assessing peer status in childhood and allows children’s peer acceptance to be interpreted relative to their immediate classroom peer group.

#### 2.3.5 Control variables

Child age (in months), gender (0 = boy, 1 = girl), and family socioeconomic status (SES) were collected via parent report. SES was computed as a composite of standardized parental education and occupational prestige scores. Child verbal ability was assessed at T1 using the Peabody Picture Vocabulary Test-Revised (PPVT-R; Dunn and Dunn, 2007) Chinese version, with raw scores used as a covariate.

### 2.4 Data analysis

Data analyses were conducted using Mplus 8.6 and SPSS 27.0. The analytical strategy proceeded in sequential steps, progressing from preliminary analyses to test our theoretical model.

First, descriptive statistics (means, standard deviations, ranges) and bivariate Pearson correlations were computed to examine the preliminary relationships among all study variables at T1 and T2. This step served to: (a) assess the basic patterns of association between key constructs (e.g., between teacher-child closeness and emotion understanding), (b) provide an initial, foundational test of the interrelationships posited in Hypothesis 0, and (c) check for the presence of high multicollinearity that might complicate subsequent modeling. Separate correlation matrices were generated for T1 variables and T2 variables. Additionally, cross-temporal correlations (e.g., between T1 and T2 measures of peer acceptance) were examined to assess the stability of constructs over the 12 months. Missing data for these preliminary analyses were handled using pairwise deletion, though all subsequent model-based analyses employed the more robust Full Information Maximum Likelihood (FIML) estimation, which produces unbiased parameter estimates under the missing-at-random assumption and uses all available data.

To test our primary longitudinal hypotheses, we employed structural equation modeling (SEM). We began by specifying a model to test the direct effects of T1 teacher-child closeness and conflict on T2 peer acceptance, while controlling for T1 peer acceptance, age, gender, verbal ability, and SES (Hypothesis 1).

Second, we tested simple mediation models in which T2 emotion understanding and T2 social initiative independently mediated the relationship between T1 teacher-child relationship and T2 peer acceptance. In these models, we controlled for the baseline (T1) level of the respective mediator and outcome variable, along with other covariates (Hypothesis 2).

Finally, to examine whether the data were consistent with the proposed theory-informed serial indirect pathway, we specified a comprehensive two-wave longitudinal path model: T1 teacher–child relationship quality → T2 emotion understanding → T2 social initiative → T2 peer acceptance. In this model, T1 emotion understanding, T1 social initiative, and T1 peer acceptance were included as baseline controls, and child age, gender, language ability, and family SES were included as covariates. Importantly, because emotion understanding, social initiative, and peer acceptance were all assessed at T2, the paths among these T2 variables were interpreted as within-wave residual associations embedded in a longitudinal framework, rather than as definitive evidence of temporal precedence. The significance of indirect effects was tested using bias-corrected bootstrap confidence intervals based on 5,000 resamples. A 95% confidence interval that did not include zero indicated a statistically significant indirect effect. Model fit was evaluated using standard indices: χ^2^/df < 3, Comparative Fit Index (CFI) > 0.95, Tucker-Lewis Index (TLI) > 0.95, Root Mean Square Error of Approximation (RMSEA) < 0.06, and Standardized Root Mean Square Residual (SRMR) < 0.08.

## 3 Results

### 3.1 Sample description

The final longitudinal sample for analysis comprised *N* = 319 preschool children. Descriptive statistics for key sociodemographic and control variables at Time 1 (T1) are presented in Table 1. Children’s mean age at T1 was 4.49 years (SD = 0.77), with an age range from 2.07 to 7.39 years. The sample was nearly evenly distributed by gender, with 154 boys (49.2%) and 159 girls (50.8%). Children’s verbal ability, as assessed by the Peabody Picture Vocabulary Test-Revised (PPVT-R) raw score, had a mean of 101.45 (SD = 16.07), ranging from 59.55 to 139.49. The composite score for family socioeconomic status (SES), standardized within the sample, had a mean near zero (*M* = 0.14, SD = 1.05), with values ranging from −2.90 to 2.53.

**TABLE 1:** Sociodemographic characteristics of the sample (*N* = 319).

Variable	Mean (SD) or n (%)	Range
Age at T1 (years)	4.49 (0.77)	2.07–7.39
Gender		
Boys	154 (49.2%)	
Girls	159 (50.8%)	
Language Ability (PPVT-R)	101.45 (16.07)	59.55–139.49
Family SES (standardized)	0.14 (1.05)	−2.90 to 2.53

PPVT-R, Peabody Picture Vocabulary Test-Revised; SES, Socioeconomic Status; T1, Time 1.

Table 2 and Figure 1 presents the descriptive statistics and bivariate Pearson correlations among all study variables across the two time points (*N* = 319 pairs of observations). The means and standard deviations for the core variables are as follows. At Time 1 (T1), Teacher Closeness averaged 3.05 (SD = 0.73), Teacher Conflict averaged 1.96 (SD = 0.79), Emotion Understanding averaged 10.07 (SD = 4.13), Social Initiative averaged 2.86 (SD = 1.08), and Peer Acceptance averaged 1.62 (SD = 0.23). At Time 2 (T2), Emotion Understanding averaged 11.73 (SD = 4.05), Social Initiative averaged 2.14 (SD = 0.85), and Peer Acceptance averaged 1.06 (SD = 0.26). The correlation matrix revealed a consistent pattern of significant interrelationships. Teacher Closeness at T1 was positively correlated with T1 Emotion Understanding (*r* = 0.300, *p* < 0.001), T1 Social Initiative (*r* = 0.165, *p* < 0.01), T1 Peer Acceptance (*r* = 0.650, *p* < 0.001), and all corresponding T2 variables (Emotion Understanding: *r* = 0.318; Social Initiative: *r* = 0.212; Peer Acceptance: *r* = 0.591, all *p* < 0.001). Conversely, Teacher Conflict at T1 was negatively correlated with T1 Emotion Understanding (*r* = −0.271, *p* < 0.001), T1 Peer Acceptance (*r* = −-0.168, *p* < 0.01), and T2 Emotion Understanding (*r* = −0.218, *p* < 0.001). Substantial stability coefficients were observed for Emotion Understanding (*r* = 0.664, *p* < 0.001), Social Initiative (*r* = 0.531, *p* < 0.001), and Peer Acceptance (*r* = 0.722, *p* < 0.001) from T1 to T2. Furthermore, the two proposed mediators at T2 were positively correlated with each other (*r* = 0.458, *p* < 0.001) and with the T2 outcome, Peer Acceptance (Emotion Understanding: *r* = 0.514; Social Initiative: *r* = 0.605, both *p* < 0.001).

**TABLE 2:** Descriptive statistics and correlation analysis (*n* = 319).

Variables	M	SD	1	2	3	4	5	6	7	8
1. Teacher closeness (T1)	3.05	0.73	–							
2. Teacher conflict (T1)	1.96	0.79	−0.416***	–						
3. Emotion understanding (T1)	10.07	4.13	0.300***	−0.271***	–					
4. Social initiative (T1)	2.86	1.08	0.165**	−0.071	0.258***	–				
5. Peer acceptance (T1)	1.62	0.23	0.650***	−0.168**	0.427***	0.414***	–			
6. Emotion understanding (T2)	11.73	4.05	0.318***	−0.218***	0.664***	0.224***	0.364***	–		
7. Social initiative (T2)	2.14	0.85	0.212***	−0.045	0.264***	0.531***	0.335***	0.458***	–	
8. Peer acceptance (T2)	1.06	0.26	0.591***	−0.161**	0.380***	0.326***	0.722***	0.514***	0.605***	–

**p < 0.01, ***p < 0.001.

**FIGURE 1:**
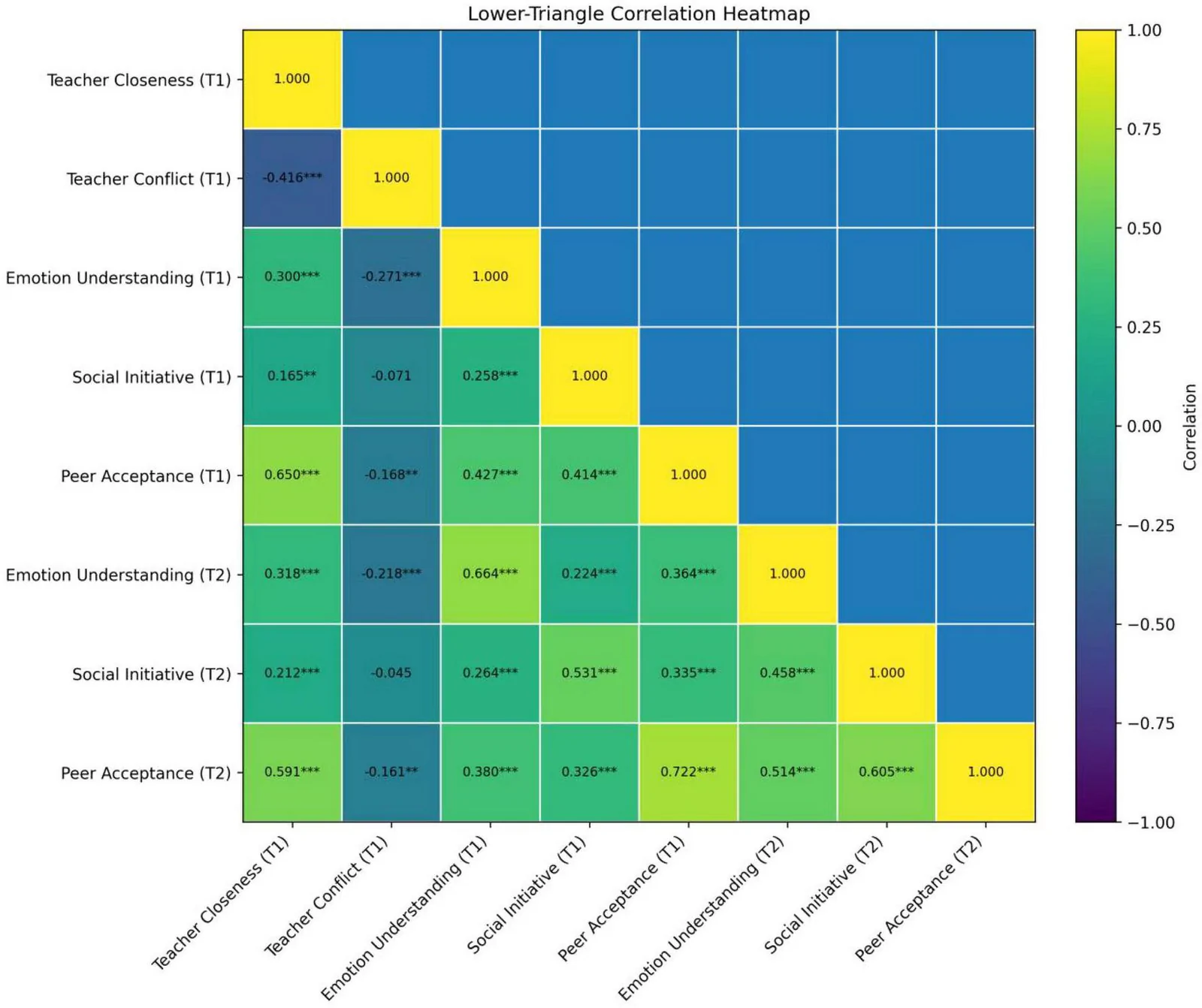
Correlation heatmap of all variables. **p < 0.01, ***p < 0.001.

### 3.2 Psychometric properties of the Social Initiative Scale

Psychometric properties of the Social Initiative Scale are presented in Table 3. Because the Social Initiative scale was developed for this study, additional psychometric analyses were conducted. The six-item scale showed good internal consistency at both waves, with Cronbach’s α = 0.860 at T1 and 0.880 at T2. Confirmatory factor analyses supported a one-factor structure. The model fit was acceptable at T1, χ^2^(9) = 8.06, χ^2^/df = 0.90, CFI = 1.000, TLI = 1.001, RMSEA = 0.000, SRMR = 0.003, and at T2, χ^2^(9) = 10.90, χ^2^/df = 1.21, CFI = 0.999, TLI = 0.999, RMSEA = 0.027, SRMR = 0.005. Standardized factor loadings ranged from 0.745 to 0.859 at T1 and from 0.736 to 0.833 at T2. Composite reliability was 0.898 at T1 and 0.890 at T2, and average variance extracted was 0.595 at T1 and 0.575 at T2. Social initiative was significantly correlated with emotion understanding at T1, *r* = 0.258, *p* < 0.001, and T2, *r* = 0.224, *p* < 0.001, and with peer acceptance at T1, *r* = 0.414, *p* < 0.001, and T2, *r* = 0.326, *p* < 0.001. These results indicate that the Social Initiative scale demonstrated acceptable internal consistency, one-factor structure, and convergent validity in the present sample, while showing associations with related but non-identical constructs.

**TABLE 3:** Psychometric properties of the Social Initiative Scale.

Item	Item description	Loading T1	Loading T2
SI-1	Invites other children to play	0.755	0.745
SI-2	Starts conversations with peers during free play	0.758	0.744
SI-3	Actively joins group play in an appropriate way	0.745	0.751
SI-4	Suggests play ideas to peers	0.752	0.738
SI-5	Approaches peers positively when entering play	0.753	0.736
SI-6	Maintains active engagement in peer activities	0.859	0.833
Cronbach’s α		0.860	0.880
Composite reliability		0.898	0.595
Average variance extracted		0.890	0.575

### 3.3 Longitudinal changes in key variables

Paired-sample *t*-tests were conducted to examine mean-level changes in the key mediator and outcome variables from Time 1 (T1) to Time 2 (T2). The results are presented in Table 4 and Figure 2. A significant increase was observed in Emotion Understanding scores from T1 (*M* = 10.07, SD = 4.13) to T2 (*M* = 11.73, SD = 4.05), *t* = 6.42, *p* < 0.001. The effect size was small-to-medium (Cohen’s *d* = 0.41, 95% CI [0.28, 0.54]). In contrast, significant decreases were found for both Social Initiative and Peer Acceptance. Social Initiative scores declined from T1 (*M* = 2.86, SD = 1.08) to T2 (*M* = 2.14, SD = 0.85), *t* = 4.18, *p* < 0.001, with a small effect size (Cohen’s *d* = 0.27, 95% CI [0.14, 0.40]). Peer Acceptance scores also declined from T1 (*M* = 1.62, SD = 0.23) to T2 (*M* = 1.06, SD = 0.26), *t* = 2.95, *p* = 0.003, with a small effect size (Cohen’s *d* = 0.19, 95% CI [0.06, 0.32]).

**TABLE 4:** Descriptive statistics and paired-sample *t*-tests for key variables.

Variable	Time Point	Mean	SD	*t*-value	*p*-value	Cohen’s *d* [95% CI]
Emotion Understanding	T1	10.07	4.13	6.42	<0.001	0.41 [0.28, 0.54]
	T2	11.73	4.05			
Social initiative	T1	2.86	1.08	4.18	<0.001	0.27 [0.14, 0.40]
	T2	2.14	0.85			
Peer acceptance	T1	1.62	0.23	2.95	0.003	0.19 [0.06, 0.32]
	T2	1.06	0.26			

Effect size interpretation: small (0.2), medium (0.5), large (0.8). All tests are two-tailed.

**FIGURE 2:**
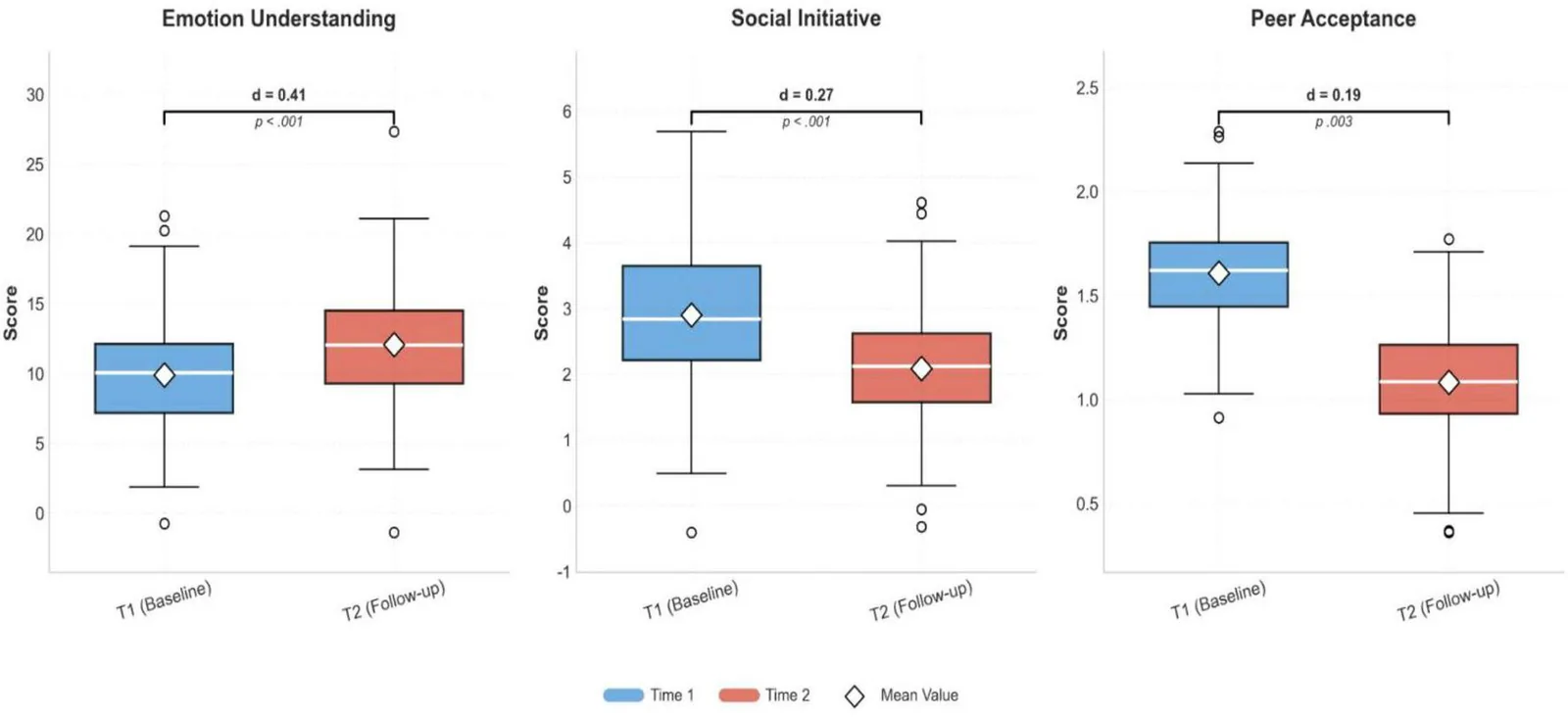
Longitudinal comparison box plot of key variables.

### 3.4 Classroom-level clustering

Because children were nested within 12 classrooms, intraclass correlation coefficients were calculated for the main study variables. The results are presented in Table 5. The ICCs ranged from 0.019 to 0.068. Specifically, the ICC was 0.019 for teacher closeness and 0.043 for teacher conflict. For the T1 child-level variables, the ICCs were 0.053 for emotion understanding, 0.021 for social initiative, and 0.042 for peer acceptance. For the T2 variables, the ICCs were 0.052 for emotion understanding, 0.068 for social initiative, and 0.061 for peer acceptance. The corresponding design effects ranged from 1.48 to 2.75. Several variables, including teacher conflict, emotion understanding, peer acceptance, and T2 social initiative, had design effects greater than 2.00.

**TABLE 5:** Intraclass correlation coefficients for main study variables.

Variable	ICC	Average classroom size	Design effect	Interpretation
Teacher closeness T1	0.019	26.58	1.48	Limited clustering
Teacher conflict T1	0.043	26.58	2.09	Non-negligible clustering
Emotion understanding T1	0.053	26.58	2.36	Non-negligible clustering
Social initiative T1	0.021	26.58	1.55	Limited clustering
Peer acceptance T1	0.042	26.58	2.08	Non-negligible clustering
Emotion understanding T2	0.052	26.58	2.33	Non-negligible clustering
Social initiative T2	0.068	26.58	2.75	Non-negligible clustering
Peer acceptance T2	0.061	26.58	2.57	Non-negligible clustering

ICC, intraclass correlation coefficient. Design effect was calculated as 1 + (average classroom size − 1) × ICC. The average classroom size was 26.58 children.

Accordingly, the main structural models were re-estimated using cluster-robust standard errors with classroom specified as the clustering variable. The substantive pattern of findings remained unchanged after this correction. The directions and statistical significance of the main prospective paths and indirect pathways were consistent with the original models.

### 3.5 Structural equation modeling

A structural equation model was tested to examine the direct longitudinal effects of T1 teacher-child relationship quality on T2 peer acceptance (show in Table 6), while controlling for T1 peer acceptance and covariates (Hypothesis 1). The model indicate good fit to the data: χ^2^/df = 2.18, Comparative Fit Index (CFI) = 0.96, Tucker-Lewis Index (TLI) = 0.94, Root Mean Square Error of Approximation (RMSEA) = 0.061 (90% CI: 0.045, 0.077), Standardized Root Mean Square Residual (SRMR) = 0.039. The standardized path coefficients are presented in Table 5. After accounting for the stability of peer acceptance (β = 0.52, *p* < 0.001), T1 Teacher Closeness showed a significant positive direct effect on T2 Peer Acceptance (β = 0.18, *p* = 0.003). Conversely, T1 Teacher Conflict showed a significant negative direct effect on T2 Peer Acceptance (β = −0.21, *p* < 0.001). Among the control variables, T1 Language Ability had a small but significant positive effect on T2 Peer Acceptance (β = 0.11, *p* = 0.028). The effects of T1 Age (β = 0.08, *p* = 0.110), Gender (β = 0.04, *p* = 0.424), and T1 SES (β = 0.09, *p* = 0.072) on T2 Peer Acceptance were not statistically significant.

**TABLE 6:** Model fit indices and standardized direct effects.

Path	β	SE	*z*	*p*	95% CI
T1 teacher closeness → T2 peer acceptance	0.18	0.06	3.00	0.003	[0.06, 0.30]
T1 teacher conflict → T2 peer acceptance	−0.21	0.06	−3.50	<0.001	[−0.33, −0.09]
T1 peer acceptance → T2 peer acceptance	0.52	0.05	10.40	<0.001	[0.42, 0.62]
T1 age → T2 peer acceptance	0.08	0.05	1.60	0.110	[−0.02, 0.18]
Gender → T2 peer acceptance	0.04	0.05	0.80	0.424	[−0.06, 0.14]
T1 language ability → T2 peer acceptance	0.11	0.05	2.20	0.028	[0.01, 0.21]
T1 SES → T2 peer acceptance	0.09	0.05	1.80	0.072	[−0.01, 0.19]

Model Fit: χ^2^/df = 2.18, CFI = 0.96, TLI = 0.94, RMSEA = 0.061 (90% CI: 0.045, 0.077), SRMR = 0.039. CI, confidence interval.

Separate structural equation models were tested to examine whether T2 Emotion Understanding (Model A) and T2 Social Initiative (Model B) independently mediated the longitudinal association between T1 teacher-child relationship quality and T2 peer acceptance (Hypothesis 2) (show in Table 7 and Figure 3). Both models controlled for the respective T1 levels of the mediator and outcome variable, age, gender, language ability, and SES. The fit indices for both models were good (CFI > 0.95, TLI > 0.94, RMSEA < 0.06, SRMR < 0.05). The results are detailed in Table 6. In Model A, T1 Teacher Closeness positively predicted T2 Emotion Understanding (β = 0.15, *p* = 0.012), and T1 Teacher Conflict negatively predicted T2 Emotion Understanding (β = −0.19, *p* = 0.002). T2 Emotion Understanding, in turn, positively predicted T2 Peer Acceptance (β = 0.21, *p* < 0.001). The specific indirect effect from Teacher Closeness to Peer Acceptance via Emotion Understanding was significant (β = 0.032, 95% BC Bootstrap CI [0.006, 0.058]). Similarly, the indirect effect from Teacher Conflict to Peer Acceptance via Emotion Understanding was also significant (β = −0.040, 95% BC Bootstrap CI [−0.068, −0.012]). In Model B, T1 Teacher Closeness positively predicted T2 Social Initiative (β = 0.17, *p* = 0.005), and T1 Teacher Conflict negatively predicted T2 Social Initiative (β = −0.22, *p* < 0.001). T2 Social Initiative positively predicted T2 Peer Acceptance (β = 0.28, *p* < 0.001). The specific indirect effect from Teacher Closeness to Peer Acceptance via Social Initiative was significant (β = 0.048, 95% BC Bootstrap CI [0.018, 0.078]). The indirect effect from Teacher Conflict to Peer Acceptance via Social Initiative was also significant (β = −0.062, 95% BC Bootstrap CI [−0.095, −0.029]).

**TABLE 7:** Simple mediation models testing independent pathways.

Path	β	SE	*z*	*p*	95% CI
Model A: T2 emotion understanding as mediator					
T1 teacher closeness → T2 emotion understanding	0.15	0.06	2.50	0.012	[0.03, 0.27]
T1 teacher conflict → T2 emotion understanding	−0.19	0.06	−3.17	0.002	[−0.31, −0.07]
T2 emotion understanding → T2 peer acceptance	0.21	0.06	3.50	<0.001	[0.09, 0.33]
Specific indirect effects					
Closeness → emotion understanding → acceptance	0.032	0.013	–	–	[0.006, 0.058]
Conflict → emotion understanding → acceptance	−0.040	0.014	–	–	[−0.068, −0.012]
Model B: T2 social initiative as mediator					
T1 teacher closeness → T2 social initiative	0.17	0.06	2.83	0.005	[0.05, 0.29]
T1 teacher conflict → T2 social initiative	−0.22	0.06	−3.67	<0.001	[−0.34, −0.10]
T2 social initiative → T2 peer acceptance	0.28	0.06	4.67	<0.001	[0.16, 0.40]
Specific indirect effects					
Closeness → social initiative → acceptance	0.048	0.015	–	–	[0.018, 0.078]
Conflict → social initiative → acceptance	−0.062	0.017	–	–	[−0.095, −0.029]

Both models controlled for the respective T1 level of the mediator and outcome variable, age, gender, language ability, and SES. Bias-corrected bootstrap confidence intervals (CIs) are based on 5,000 resamples. CIs not containing zero indicate significance. Both models showed good fit (CFI > .95, TLI > .94, RMSEA < .06, SRMR < .05).

**FIGURE 3:**
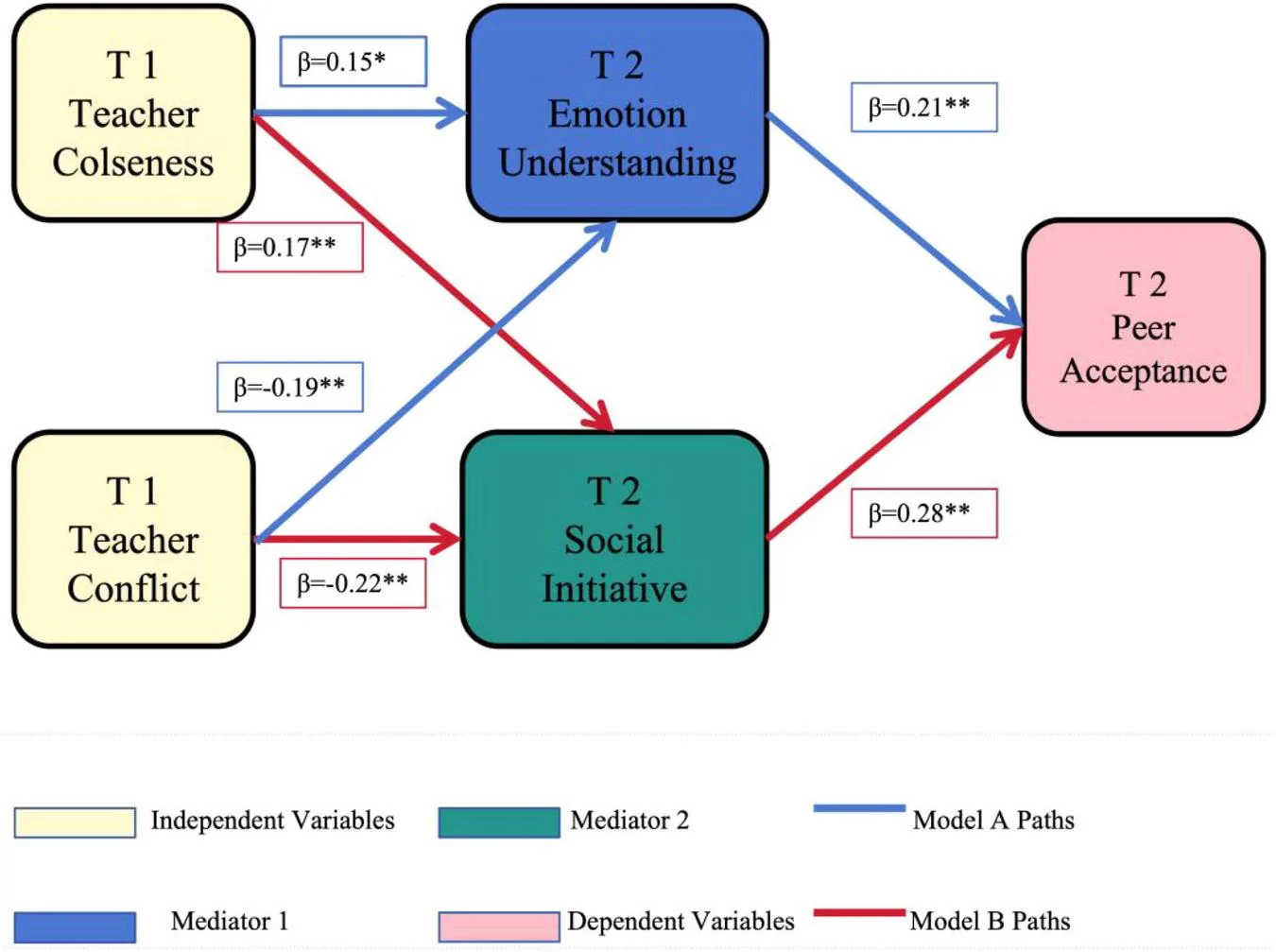
Two-mediation model path diagram. **p < 0.01, ***p < 0.001.

To examine whether the data were consistent with the theory-informed serial indirect pathway proposed in Hypothesis 3, a comprehensive two-wave longitudinal path model was specified. The model estimated the pathway from T1 teacher–child relationship quality to T2 peer acceptance through T2 emotion understanding and T2 social initiative, while controlling for the corresponding T1 baseline levels of emotion understanding, social initiative, and peer acceptance, as well as age, gender, language ability, and SES. The model indicate adequate fit to the data: χ^2^/df = 2.31, CFI = 0.95, TLI = 0.93, RMSEA = 0.064, SRMR = 0.042.

The standardized direct path coefficients and indirect effect estimates are presented in Table 8 and Figure 4. The direct paths within the proposed sequence were all significant: T1 Teacher Closeness positively predicted T2 Emotion Understanding (β = 0.14, *p* = 0.020), and T1 Teacher Conflict negatively predicted it (β = −0.18, *p* = 0.003). T2 Emotion Understanding positively predicted T2 Social Initiative (β = 0.33, *p* < 0.001), which in turn positively predicted T2 Peer Acceptance (β = 0.24, *p* < 0.001).

**TABLE 8:** Theory-informed serial indirect path model: path coefficients and indirect effects.

Path	β /Estimate	SE	*z*	*p*	95% CI/Bootstrap CI
Direct paths					
T1 teacher closeness → T2 emotion understanding	0.14	0.06	2.33	0.020	[0.02, 0.26]
T1 teacher conflict → T2 emotion understanding	−0.18	0.06	−3.00	0.003	[−0.30, −0.06]
T2 emotion understanding → T2 social initiative	0.33	0.06	5.50	<0.001	[0.21, 0.45]
T2 social initiative → T2 peer acceptance	0.24	0.06	4.00	<0.001	[0.12, 0.36]
T1 teacher closeness → T2 peer acceptance (direct)	0.11	0.06	1.83	0.067	[−0.01, 0.23]
T1 teacher conflict → T2 peer acceptance (direct)	−0.14	0.06	−2.33	0.020	[−0.26, −0.02]
Indirect effects					
Closeness → emotion understanding → social initiative	0.011	0.005	–	–	[0.002, 0.022]
Closeness → emotion understanding → social initiative → acceptance	0.011	0.004	–	–	[0.004, 0.020]
Closeness → social initiative → acceptance	0.041	0.013	–	–	[0.015, 0.067]
Total Indirect effect (closeness)	0.063	0.018	–	–	[0.028, 0.098]
Conflict → emotion understanding → social initiative	−0.014	0.006	–	–	[−0.027, −0.003]
Conflict → emotion understanding → social initiative → acceptance	−0.014	0.006	–	–	[−0.027, −0.004]
Conflict → social initiative → acceptance	−0.053	0.016	–	–	[−0.085, −0.022]
Total indirect effect (conflict)	−0.081	0.022	–	—	[−0.124, −0.038]

Model Fit: χ^2^/df = 2.31, CFI = 0.95, TLI = 0.93, RMSEA = 0.064 (90% CI: 0.050, 0.078), SRMR = 0.042. Note. Direct paths report standardized coefficients (β), standard errors (SE), z-values, *p*-values, and 95% confidence intervals (CI). Indirect effects report standardized estimates (β), standard errors (SE), and 95% bias-corrected bootstrap confidence intervals (based on 5,000 resamples). For indirect effects, *z*-values and *p*-values are not reported as significance is determined by bootstrap confidence intervals that do not contain zero.

**FIGURE 4:**
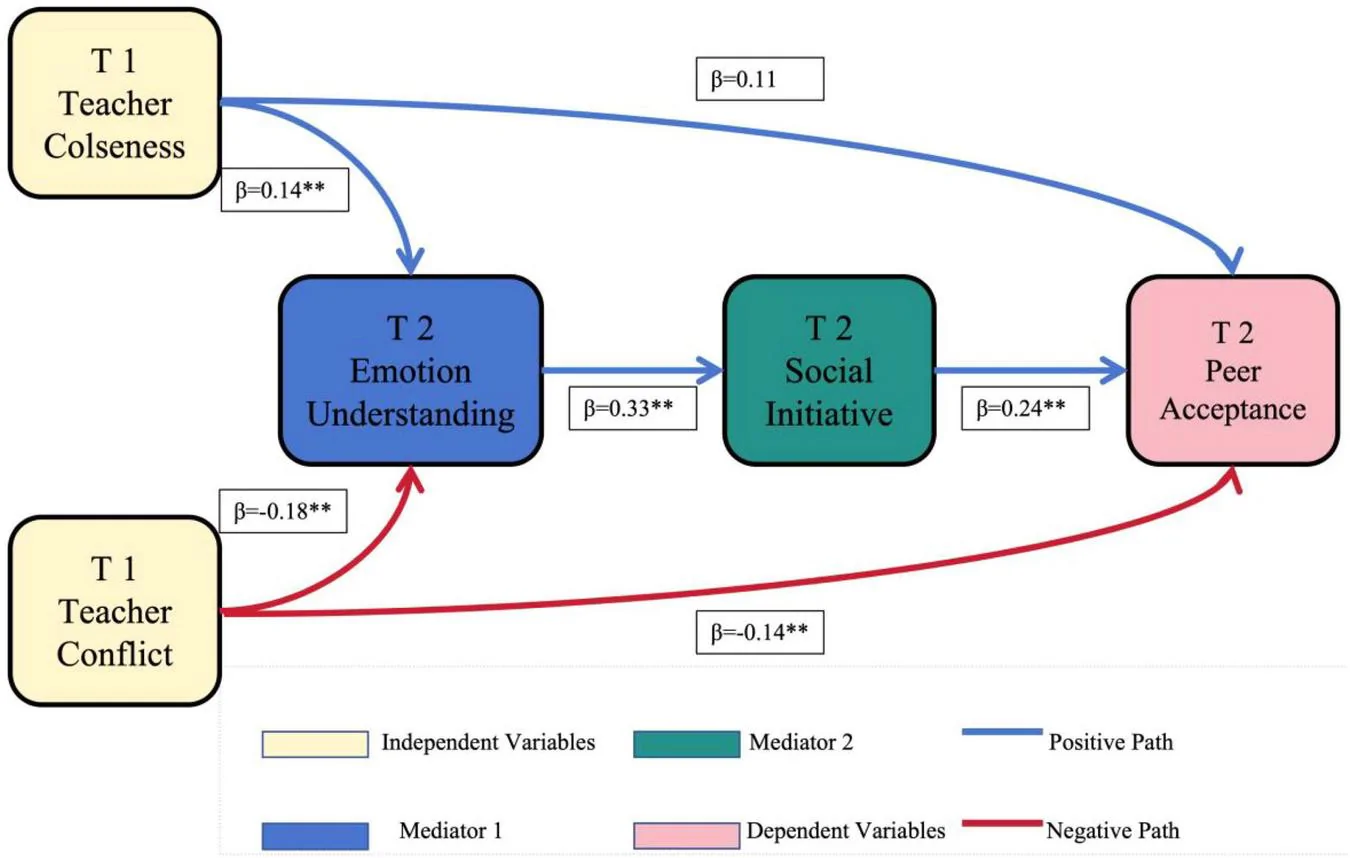
Theory-informed serial indirect path model. **p < 0.01.

When the mediators were included in the model, the direct effect of T1 Teacher Closeness on T2 Peer Acceptance was reduced and became marginally significant (β = 0.11, *p* = 0.067, 95% CI [−0.01, 0.23]). The direct effect of T1 Teacher Conflict on T2 Peer Acceptance remained significant but was attenuated (β = −0.14, *p* = 0.020).

The key specific indirect effects were tested using bias-corrected bootstrap confidence intervals based on 5,000 resamples. The sequential indirect pathway from T1 Teacher Closeness to T2 Peer Acceptance via T2 Emotion Understanding and then T2 Social Initiative was significant (β = 0.011, 95% BC Bootstrap CI [0.004, 0.020]). The corresponding sequential indirect pathway from T1 Teacher Conflict was also significant (β = −0.014, 95% BC Bootstrap CI [−0.027, −0.004]). Other significant indirect paths included the two-path mediated effects (e.g., Closeness → EU → SI; Closeness → SI → Acceptance; Conflict → SI → Acceptance). The total indirect effect was significant for both Closeness (β = 0.063, 95% BC Bootstrap CI [0.028, 0.098]) and Conflict (β = −0.081, 95% BC Bootstrap CI [−0.124, −0.038]).

## 4 Discussion

This two-wave longitudinal study examined whether teacher–child relationship quality was prospectively associated with preschool children’s peer acceptance across a 12-month period and whether emotion understanding and social initiative were implicated in this association. Grounded in attachment theory, social information-processing perspectives, and performance-based models of social competence, the study tested a theory-informed indirect path model linking T1 teacher–child closeness and conflict with T2 peer acceptance through T2 emotion understanding and T2 social initiative. Overall, the findings were consistent with the proposed conceptual model. At the same time, the observational design and the same-wave assessment of the mediators and outcome require caution. The results should therefore be interpreted as prospective and theory-informed associations rather than as evidence of causal or strictly temporal developmental processes.

### 4.1 Synthesis of key findings and hypothesis support

The findings provided multi-layered support for the hypothesized pattern of associations. At the foundational level, the correlation analyses showed significant links among teacher–child relationship quality, emotion understanding, social initiative, and peer acceptance, both concurrently and across time. Teacher closeness was positively associated with children’s emotion understanding, social initiative, and peer acceptance, whereas teacher conflict generally showed negative associations with these indicators. This pattern is consistent with previous research suggesting that children’s relationships with teachers are meaningfully related to socio-emotional functioning and peer adjustment.

The structural models further showed that teacher–child relationship quality at T1 prospectively predicted peer acceptance at T2 after baseline peer acceptance and relevant covariates were controlled. Specifically, teacher closeness was positively associated with later peer acceptance, whereas teacher conflict was negatively associated with later peer acceptance. These findings extend prior cross-sectional evidence by indicating that teacher–child relationship quality is related to residualized change in peer acceptance over time. However, because the study was observational, these results should not be interpreted as evidence that teacher–child relationship quality causally determines later peer acceptance. The attenuated direct paths in the final model also suggest that the proposed indirect pathways do not exhaust the possible mechanisms involved; other factors, such as general self-esteem and classroom-level processes, may also contribute to children’s peer adjustment (Scherrer et al., 2023; Morin et al., 2013).

The independent indirect pathway analyses suggested two related but distinguishable processes. The pathway through emotion understanding is consistent with the view that teachers may function as important emotion socializers in early childhood classrooms. The pathway through social initiative highlights the relevance of children’s proactive behavioral engagement with peers. The findings also provided qualified support for the theory-informed serial indirect pathway. Specifically, teacher–child relationship quality was associated with later peer acceptance through a pattern involving emotion understanding and social initiative. This pattern is consistent with the theoretical proposition that children with stronger emotion understanding may be better positioned to engage peers proactively, which in turn may be associated with greater peer acceptance. Nevertheless, this interpretation must be approached cautiously. Emotion understanding, social initiative, and peer acceptance were all assessed at T2; therefore, the present design cannot empirically demonstrate that emotion understanding preceded social initiative, or that social initiative preceded peer acceptance. The serial pathway should be understood as a theoretically plausible pattern of associations within a two-wave longitudinal framework rather than as confirmation of a definitive developmental sequence.

The observed mean-level decreases in social initiative and peer acceptance from T1 to T2 also deserve attention. These decreases do not necessarily indicate a simple decline in children’s social functioning; rather, they may reflect developmental and contextual changes that occur as preschool children move through a more complex peer ecology. As children grow older, peer groups often become more differentiated, social entry becomes more contingent on established friendships and group norms, and teachers may apply higher expectations when rating proactive peer engagement. In this context, lower average social initiative may indicate that children become more selective or cautious in initiating interactions, whereas lower peer acceptance may reflect increasing differentiation in peer preferences rather than a uniform deterioration in peer relationships.

These mean-level patterns are important because they suggest that later preschool years may represent a period in which children face increasing social demands. Even when emotion understanding improves, children may still require support in translating socio-emotional knowledge into effective peer-entry behaviors. Therefore, the decline in social initiative and peer acceptance provides an additional developmental context for interpreting the indirect pathway: children’s proactive engagement with peers may become especially important when peer networks become more selective and socially demanding.

It is also important to place these findings within a broader developmental framework. Although the model focused on emotion understanding and social initiative, these constructs do not operate in isolation. General cognitive ability, attentional functioning, self-regulation, and executive functions may simultaneously shape teacher–child relationship quality, emotion understanding, social initiative, and peer acceptance. Recent evidence further supports this broader interpretation. Commodari et al. (2026), in a study of preschool children, found that visual focused attention, visual–spatial focused attention, and short-term attentional span were consistently associated with global and domain-specific learning outcomes, while the contribution of divided attention varied according to children’s social functioning profiles. Their findings suggest that early learning competence reflects the interplay between attentional capacities and the social contexts in which such capacities are expressed. This perspective helps contextualize the present results: social initiative and peer acceptance may reflect not only relational and emotional processes but also broader attentional and learning-related competencies that support children’s participation in structured classroom and peer activities.

### 4.2 Theoretical implications: a theory-informed pathway within a broader developmental system

The present findings have several theoretical implications for understanding early social development. First, the results are consistent with theoretical perspectives that position the teacher–child relationship as an important relational context for children’s social adaptation. Attachment-oriented accounts emphasize the secure-base function of the teacher and suggest that warm and low-conflict relationships may provide children with relational confidence in the classroom (Al-Yagon and Mikulincer, 2006). In the present study, teacher closeness and conflict were prospectively associated with peer acceptance, indicating that children’s vertical relationships with teachers are meaningfully linked to their horizontal relationships with peers.

Second, the findings connect this relational perspective with social information-processing models. Emotion understanding reflects children’s capacity to identify, interpret, and reason about emotional cues in social situations. Such capacities are relevant to peer interaction because children must infer others’ feelings, intentions, and likely responses during play, cooperation, and conflict. In this respect, the findings align with social information-processing frameworks emphasizing the importance of cue interpretation in children’s social adjustment (Crick and Dodge, 1996). However, the results should not be taken to mean that emotion understanding alone is sufficient for peer acceptance. Rather, emotion understanding was associated with social initiative, suggesting that socio-cognitive skills may be most relevant to peer adjustment when they are accompanied by active engagement in peer contexts.

Third, the results speak to the distinction between social competence and social performance. Performance-based models emphasize that possessing a social skill is not equivalent to using that skill effectively in real interactional settings (Odom and McConnell, 1985). The present findings are consistent with this distinction. Emotion understanding may represent one aspect of children’s socio-cognitive competence, whereas social initiative reflects the behavioral tendency to approach peers, invite them into play, initiate conversation, and participate actively in peer activities. This interpretation also accords with the competence-performance distinction discussed in earlier work (Wood and Power, 1987). Thus, peer acceptance may be better understood by considering both children’s understanding of social-emotional information and their behavioral participation in peer interaction.

Fourth, the findings should be interpreted within integrative developmental models that link relational, regulatory, attentional, and learning-related processes. Commodari et al. (2026) emphasized that preschool social functioning is embedded in adult-child interaction contexts and is closely associated with attention and learning-related competencies. This evidence is relevant to the present study because social initiative requires children to monitor social cues, divide attention between ongoing play and peer responses, and adjust behavior to classroom expectations. Peer acceptance may likewise depend on a broader set of capacities that includes emotional understanding, behavioral regulation, attention, and learning-related engagement. Accordingly, the present model should be viewed as one theoretically specified pathway within a wider developmental system rather than as a closed or exclusive mechanism.

This broader interpretation is particularly important because children were nested within classrooms. Classroom climate, teacher practices, peer norms, and group composition may shape how social initiative is expressed and how peer acceptance develops. The present findings therefore contribute to a more integrated understanding of preschool peer adjustment while also highlighting the need for future research that models child-level, relational, attentional, and classroom-level processes together.

### 4.3 Practical implications for early childhood education and intervention

The magnitude of the observed effects should also be considered when interpreting the practical significance of the findings. Although several paths were statistically significant, most standardized estimates were small to moderate in size. This pattern is common in longitudinal developmental studies, particularly when baseline levels and covariates are controlled. The findings therefore should not be interpreted as indicating large or deterministic effects of teacher–child relationship quality, emotion understanding, or social initiative on peer acceptance. Rather, they suggest that these factors are modest but potentially meaningful contributors to preschool peer adjustment. From a practical perspective, this means that interventions targeting teacher–child relationships, emotion understanding, and social initiative may be useful, but they are unlikely to be sufficient on their own. Such efforts should be embedded within broader classroom practices that also support attention, self-regulation, cooperative play, and inclusive peer climates.

The findings offer several practical implications for early childhood education, although these implications should be viewed as theory-informed rather than causal recommendations. First, the results underscore the potential relevance of warm and low-conflict teacher–child relationships for children’s peer adjustment. Professional development programs may benefit from helping teachers build emotionally supportive relationships with children, especially those who show early social difficulties. Such efforts may include sensitive responding, positive one-on-one interactions, emotional attunement, and constructive repair following conflict. These suggestions are consistent with intervention-oriented work emphasizing the role of supportive teacher–child relationships in children’s adjustment (Rucinski et al., 2018).

Second, the findings suggest that emotion understanding may be an important target within early childhood classrooms. Teachers can provide opportunities for children to label emotions, discuss the causes and consequences of emotions, and consider others’ perspectives during everyday classroom interactions. Storybook reading, guided conversations, and reflective discussions of peer conflict may all provide natural contexts for supporting children’s emotional understanding. Such practices are consistent with work emphasizing children’s emotional engagement with stories and classroom discourse (Alexander et al., 2001).

Third, the association between social initiative and peer acceptance suggests that children may benefit from opportunities to translate socio-emotional understanding into peer engagement. Teachers can support this process by structuring cooperative play, guiding peer entry, modeling appropriate invitations, and reinforcing positive attempts to initiate interaction. For example, a teacher may help a hesitant child ask to join a group activity or encourage children to invite classmates into ongoing play. Peer-mediated and teacher-facilitated strategies may be particularly useful for children who understand social cues but are less likely to initiate interaction spontaneously (Watkins et al., 2023; Fedewa et al., 2025).

Fourth, the integration of recent evidence on attention and learning-related competencies suggests that social-emotional support should not be separated from broader developmental support. Children’s ability to initiate peer interaction may depend partly on attentional control, behavioral organization, and engagement with adult-guided activities. Classroom practices that reduce excessive distraction, provide predictable routines, scaffold turn-taking, and support sustained engagement may therefore create conditions in which children can use both socio-emotional and attentional resources more effectively. However, because the present study was observational, intervention studies are needed to determine whether practices targeting teacher–child relationships, emotion understanding, social initiative, and attentional-regulatory competencies lead to subsequent improvements in peer acceptance.

The decreases in social initiative and peer acceptance further suggest that classroom practices should attend not only to children with persistently low social functioning but also to normative changes in peer-group dynamics over time. Teachers may need to provide continued support for peer entry, cooperative participation, and inclusive play as children progress through preschool, particularly during periods when peer groups become more stable or socially selective. Such support may help children maintain opportunities for positive interaction even when the classroom peer ecology becomes more complex.

### 4.4 Limitations and future research directions

Several limitations should be acknowledged. First, the observational two-wave design limits causal and temporal interpretation. Although baseline controls and the 12-month interval strengthen the prospective nature of the analyses, they do not establish causality. In addition, emotion understanding, social initiative, and peer acceptance were all assessed at T2. Thus, the theory-informed serial pathway cannot demonstrate that emotion understanding occurred before social initiative or that social initiative occurred before peer acceptance. Future studies should use experimental or quasi-experimental designs and multi-wave longitudinal models, such as three- or four-wave designs, to examine temporal ordering, reciprocal pathways, and developmental cascade processes more rigorously.

Second, teacher–child relationship quality was assessed only at T1. This approach was consistent with the aim of examining the initial relational context as a prospective predictor, but it does not capture the dynamic nature of teacher–child relationships over the preschool year. Children’s social behavior and peer acceptance may also shape later teacher perceptions and interactions. Future studies should measure teacher–child relationship quality at multiple waves and apply cross-lagged, random-intercept cross-lagged, or latent change score models to clarify reciprocal developmental processes.

Third, measurement issues should be considered. Although the study used multiple methods, including teacher reports, child assessments, and peer nominations, teacher–child relationship quality and social initiative were both reported by teachers, which may have increased shared method variance. In addition, the Social Initiative scale was developed for this study. The revised analyses provide preliminary evidence regarding its internal consistency, one-factor structure, and construct relevance, but further validation is needed. Future research should include direct observations of peer interaction, multi-informant ratings, test-retest reliability, and comparisons with established measures of broader social competence.

Fourth, the model did not directly assess several broader developmental competencies that may provide alternative explanations for the observed associations. General cognitive ability, attentional functioning, inhibitory control, working memory, self-regulation, and learning-related behaviors may influence how children interact with teachers, understand emotions, initiate peer contact, and become accepted by peers. Although age, language ability, gender, SES, and baseline variables were controlled, these covariates do not fully capture attentional and executive-functioning processes. Future studies should incorporate direct measures of attention, executive function, and self-regulation to examine whether the proposed indirect pathways remain robust after accounting for these broader developmental factors.

Fifth, the nested classroom structure and sample characteristics limit the scope of inference. Children were recruited from 12 classrooms, and classroom-level factors such as teacher practices, classroom climate, peer norms, and group composition may have shaped the study variables. The revised analyses accounted for clustering using intraclass correlation coefficients and cluster-robust standard errors; however, the number of classrooms was too small for a full multilevel structural equation model. The sample also included a relatively broad preschool age range, and although age and language ability were controlled, developmental heterogeneity may still have influenced the findings. Future research should recruit larger samples across more classrooms and consider narrower age cohorts or age-stratified designs.

The observed decreases in social initiative and peer acceptance should also be interpreted cautiously. The present study did not directly assess changes in classroom composition, friendship stability, teacher expectations, or peer-group norms across the 12-month interval. Therefore, the mean-level declines may reflect a combination of developmental change, classroom context, and measurement conditions. Future studies should examine whether these decreases are replicated in other preschool samples and whether they vary according to classroom transitions, peer-network structure, or children’s initial social status.

Finally, cultural context should be considered. The study was conducted in Chinese preschool classrooms, where teacher authority, classroom organization, and group harmony may shape the meaning of teacher closeness, teacher conflict, social initiative, and peer acceptance. Cross-cultural replications are needed to examine whether the observed associations generalize to other educational systems and cultural contexts. Taken together, these limitations suggest that the present findings should be interpreted as evidence for a theoretically meaningful set of prospective associations, while future work should test these pathways using stronger designs, broader measurement batteries, and more diverse classroom samples.

## 5 Conclusion

In conclusion, this study extends prior work by examining how teacher–child relationship quality is associated with preschool children’s later peer acceptance through socio-emotional and behavioral mechanisms. Using a two-wave longitudinal design, we found that teacher closeness and conflict predicted residualized change in peer acceptance, and that emotion understanding and social initiative served as significant mediating mechanisms. The theory-informed serial indirect pathway was consistent with the proposed model, in which understanding of emotion is linked to greater social initiative, which in turn is associated with peer acceptance. However, because the mediators and outcome were assessed at the same follow-up wave, the present findings should not be interpreted as demonstrating a definitive temporal sequence. Future multi-wave studies are needed to test this developmental ordering more rigorously. Despite this limitation, the findings suggest that early childhood interventions may benefit from simultaneously strengthening teacher–child relationships, supporting children’s emotion understanding, and creating opportunities for proactive peer engagement.

## Data Availability

The raw data supporting the conclusions of this article will be made available by the authors, without undue reservation.
